# Preparation of a novel injectable *in situ*-gelling nanoparticle with applications in controlled protein release and cancer cell entrapment

**DOI:** 10.1039/c8ra06589f

**Published:** 2018-10-09

**Authors:** Min Kyung Khang, Jun Zhou, Yihui Huang, Amirhossein Hakamivala, Liping Tang

**Affiliations:** Chemistry and Biochemistry Department, University of Texas at Arlington Arlington Texas USA; Bioengineering Department, University of Texas at Arlington P. O. Box 19138 Arlington Texas 76019-0138 USA ltang@uta.edu; Department of Biomedical Science and Environmental Biology, Kaohsiung Medical University Kaohsiung 807 Taiwan

## Abstract

Temperature sensitive injectable hydrogels have been used as drug/protein carriers for a variety of pharmaceutical applications. Oligo(ethylene glycol) methacrylate (OEGMA) monomers with varying ethylene oxide chain lengths have been used for the synthesis of *in situ* forming hydrogel. In this study, a new series of thermally induced gelling hydrogel nanoparticles (PMOA hydrogel nanoparticles) was developed by copolymerization with di(ethylene glycol) methyl ether methacrylate (MEO_2_MA), poly(ethylene glycol) methyl ether methacrylate (300 g mol^−1^, OEGMA_300_), and acrylic acid (AAc). The effects of acrylic acid content on the physical, chemical, and biological properties of the nanoparticle-based hydrogels were investigated. Due to its high electrostatic properties, addition of AAc increases LCST as well as gelation temperature. Further, using Cy5-labelled bovine serum albumin and erythropoietin (Epo) as model drugs, studies have shown that the thermogelling hydrogels have the ability to tune the release rate of these proteins *in vitro*. Finally, the ability of Epo releasing hydrogels to recruit prostate cancer cells was assessed *in vivo*. Overall, our results support that this new series of thermally induced gelling systems can be used as protein control releasing vehicles and cancer cell traps.

## Abstract 2

At body temperature, thermosensitive nanoparticles release erythropoietin to lure metastatic cancer cells.

## Graphical abstract

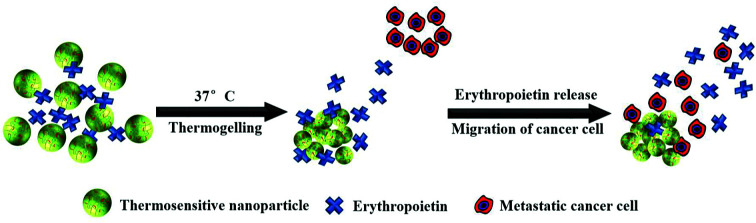

## 1. Introduction

Three-dimensional (3D) injectable hydrogel scaffolds have been widely used in cell therapy and tissue regeneration based on their unique characteristics, including controlled porosity, high water content, and mimicking of the microenvironment of natural extracellular matrices.^1,2^ Although the 3D porous scaffolds can be either formed *in situ* or preformed, the injectable *in situ* forming scaffolds offer many advantages over preformed ones. Specifically, cells and bioactive molecules can be readily incorporated into the *in situ* forming matrix by simply mixing prior to solidification. In addition, *in situ* forming gels can be implanted *via* a needle injection unlike implantation of preformed scaffolds that often require costly surgical procedures with a risk of complications.^3^ Last but not least, *in situ* forming scaffolds, but not preformed gel, can easily fill irregularly-shaped defects which are associated with different injuries and trauma.^3,4^ Consequently, there has been an increasing focus on the development of injectable *in situ*-forming systems for biomedical applications in recent years. Based on their gelation mechanisms, *in situ* forming hydrogel scaffolds can be categorized into chemically- and physically-crosslinked scaffolds.^5^ Chemically-crosslinked hydrogel scaffolds can be formed by either *in situ* polymerization or crosslinking reactions between the components. However, the toxicity and reactive nature of chemical reagents used in scaffold fabrication may adversely affect the survival and bioactivity of seeded cells and bioactive molecules.^6^ To overcome such shortcomings, *in situ* physical-crosslinking mechanisms in response to certain environmental changes such as pH, temperature, ion concentration, solvent and light, have gained in popularity.^7–12^

Among all *in situ* gelling systems, thermally induced gelling mechanism is the most common method to produce *in situ* forming scaffolds. Typically, these gel systems can flow at room temperature but solidify immediately and form 3D scaffolds when brought to body temperature (37 °C). There are two benefits of using thermally induced gel in biomedical applications. Firstly, toxic reagents and crosslinkers are not required for the production of many thermally induced gelling systems. Secondly, thermally induced gelling system can be made of a wide variety of biomaterials including natural polymers and their derivatives, synthetic polymers and polypeptides.^8–16^ While many of these thermally induced gelling systems were composed of linear or branched polymers, thermosensitive nano-/micro-size polymer particles have recently been used as building blocks for *in situ* forming hydrogel scaffolds. Compared to these linear/branched polymer-based thermogelling systems, the particle-based ones have several advantages including the reduced viscosity and improved mechanical properties at the same concentration. Furthermore, the building blocks (particles) can be employed as carriers of growth factors or bioactive molecules to deliver them in a controlled manner for guiding differentiation of stem cells.^13,17,18^ Among these particle-based thermogelling systems, poly(*N*-isopropylacrylamide) (PNIPAM)-based microgels are the most studied and have been widely explored as *in situ* forming scaffolds for use in tissue engineering.^13,17,19–26^ PNIPAM polymer has a lower critical solution temperature (LCST) at near to physiological temperature therefore, PNIPAM-based microgels can be used for reversible cell adhesion or detachment and for triggered release of therapeutics.^24,25^ However, there are limitations for long-term application in biotechnology.^27^ The monomer of PNIPAM, *N*-isopropylacrylamide (NIPAM) is carcinogenic as well as the byproducts are neurotoxic, perhaps generated by hydrolysis of PNIPAM.^18^ To overcome these concerns, a series of thermosensitive polymers or nanoparticles based on oligo(ethylene glycol) methacrylate (OEGMA) with different ethylene glycol chain lengths have been developed.^28–31^ By copolymerization of OEGMA monomers with different length of ethylene glycol chain, the as-prepared nanoparticles have a wide range of LCST, similar to those of PNIPAM or PEG-based hydrogels.^28–31^ For instance, a copolymer hydrogel based on di(ethylene glycol) methacrylate (MEO_2_MA, *n* = 2) and OEGMA_475_ (mw = 475 g mol^−1^, *n* = 8, 9) has a volume phase transition temperature (VPTT) between 23 °C and 90 °C.^32^ These results support the overall hypothesis of this work that thermally induced gelling system can be made of OEGMA particles.

Herein, we fabricated a series of new injectable *in situ* gelling hydrogel with three monomers, di(ethylene glycol) methacrylate (MEO_2_MA), OEGMA (mw = 300 g mol^−1^), and acrylic acid (AAc). The P(MEO_2_MA-*co*-OEGMA_300_-*co*-AAc) (PMOA) hydrogel has physiochemical and biological properties similar to PNIPAM and PEG. Specifically, we have synthesized three hydrogel nanoparticles with different content of acrylic acid (0, 1, and 3 mol% of AAc.) The physiochemical and biological properties of the three hydrogels were assessed *in vitro*. Finally, the capability of this new thermally induced gelling system to release proteins and recruit circulating cancer cells was assessed *in vivo*.

## 2. Materials and methods

### 2.1. Materials

Di(ethylene glycol) methyl ether methacrylate (MEO_2_MA), poly(ethylene glycol) methyl ether methacrylate (300 g mol^−1^, OEGMA_300_), acrylic acid (AAc), ethylene glycol dimethacrylate (EGDMA), and ammonium persulfate (APS) were purchased from Sigma-Aldrich (St Louis, Missouri). Sodium dodecyl sulfate (SDS) was obtained from Bio-Rad (Hercules, CA). Milli-Q grade deionized water was used through all experiments.

### 2.2. Synthesis of P(MEO_2_MA-*co*-OEGMA_300_-*co*-AAc) (PMOA) hydrogel nanoparticles

A series of hydrogel nanoparticles were synthesized *via* a free radical precipitation polymerization method.^18^ In brief, MEO_2_MA, OEGMA_300_, AAc, SDS, EGDMA and DI water were mixed in a 500 mL round bottle flask. The mixture was heated to 70 °C in a water bath under nitrogen purging. After 30 min, APS solution was added to the mixture to initiate polymerization. The reaction was allowed to proceed for 6 hours with magnetic stirring at 70 °C under N_2_ atmosphere. Here three batches of nanoparticles with different AAc contents (0, 1, and 3 mol%) were prepared (Table 1), and defined as PMOA0, PMOA1, and PMOA3, respectively. The above-prepared nanoparticle dispersions were purified with exhaustive dialysis (cutoff: 10 kDa) against deionized water for one week. The purified nanoparticles were concentrated and collected using a centrifuge, and then stored in a refrigerator for further use.

**Table 1:** Monomer composition in feed (moles). Where the total moles of three monomers are 0.026 moles, SDS is as a detergent and APS is as an initiator

Samples	MEO_2_MA (mol)	OEGMA_300_ (mol)	AAc (mol)	EGDMA (mol)	SDS (mol)	APS (mol)
PMOA0	0.02314	0.0026	0	0.00026	0.000139	0.000438
PMOA1	0.02288	0.0026	0.00026	0.00026	0.000139	0.000438
PMOA3	0.02236	0.0026	0.00078	0.00026	0.000139	0.000438

### 2.3. Size, polydispersity, zeta potential, and morphology of the particle

The size, polydispersity, and zeta potential of the hydrogel nanoparticles were determined using a ZetaPALS dynamic light scattering (DLS) detector (Brookhaven Instruments, Holtsville, NY).^33^ The samples at a concentration of 1 mg mL^−1^ in DI water were prepared, and sizes of these nanoparticles were determined at 24 and 37 °C, respectively. Furthermore, to observe morphology of the nanoparticles, Scanning Electron Microscope (SEM) was employed as described earlier.^34^ Briefly, a drop of the diluted particle dispersion was placed onto a glass slide cover adhered to a SEM specimen holder with the conductive tape, and then dried at ambient temperature. After sputter-coating with silver, SEM images were recorded by a Hitachi S-4800 II FE Scanning Electron Microscope.^34^

### 2.4. Conductivity measurement

Using a FP30 Conductivity meter (Mettle Toledo, Columbus, OH), carboxyl group content of the nanoparticles was determined quantitatively following a published method.^35^ 1.0 mL of HCl solution (0.01 M) was added into a 20 mL of the nanoparticle dispersion (30 mg mL^−1^), followed by stirring for 20 min. Conductivity measurement was conducted using NaOH aqueous solution (0.01 M) as a titrant. The COOH contents of these nanoparticles were calculated based on the following formulation: COOH (μmole mg^−1^) = *C* × *V*/*W*, where *C* and *V* are the concentration and used volume of NaOH solution, respectively. *W* is the solid content of the nanoparticles.

### 2.5. Turbidity test

To determine the phase transition temperature of the nanoparticles, the turbidity of the nanoparticles in DI water and phosphate buffered saline (PBS, 43.5 mM ionic strength) was respectively measured using a Beckman DU640 UV-visible spectrophotometer (Fullerton, CA).^36^ Briefly, 2.0 mL of the nanoparticle dispersion (1.0 mg mL^−1^) was prepared in a UV cuvette, and covered with a lid to keep water from evaporation during the experiments. The cuvette was placed in a water bath equipped with a temperature controller and incubated for one minute after the predetermined temperature was reached. The transmittance of the dispersion at various temperature points was recorded at 550 nm.

### 2.6. Viscosity test

The viscosity of the nanoparticle dispersions as a function of temperature was measured using an HR-2 Discovery Hybrid Rheometer (TA Instruments).^18,37^ Two flat parallel plates (25 cm in diameter) were used, and the distance between two plates was adjusted to 0.6 mm. During the experiments, a constant stress of 2 Pa and a frequency of 0.1 Hz was applied. To measure viscosity, 0.6 mL of nanoparticle dispersions (60 mg mL^−1^ in PBS) was loaded on the plate, viscosity of the samples was recorded with temperature increasing at a rate of 2 °C min^−1^ from 15 to 40 °C.

### 2.7. Water loss

The water retention of the hydrogels was determined using an established method.^38^ Briefly, 1.0 mL of the nanoparticle dispersion (60 mg mL^−1^ in PBS) was added to an Eppendorf tube, total mass (*W*_o_) of the tube and the sample was recorded. The tube was then transferred into a water bath and incubated at 37 °C for 4 hours. The lost water from the hydrogels during the incubation was discarded carefully from the top, and total mass (*W*_i_) of tube and hydrogel was weighted. The weight percentage of lost water was defined as: (*W*_o_ − *W*_i_)/*W*_o_ × 100%. To observe pore structures of the hydrogels, at the end of water loss experiments, the thermally gelling hydrogels were quickly frozen in liquid nitrogen and dried *in vacuo*. The cross sections of the hydrogels were observed under an SEM as described above.

### 2.8. *In vitro* release of protein

Bovine serum albumin (BSA) and erythropoietin (Epo) was used as a model protein drug and labelled with Cy®5 dye (Lumiprobe Co., Hunt Valley, MD) following the manufacturer's protocol. The release kinetics of Cy®5-labeled BSA/Epo was then determined as described previously.^18^ Briefly, 1.0 mL of nanoparticle dispersion (60 mg mL^−1^ in PBS) was mixed with 1.0 mg of Cy®5-labeled BSA/Epo at room temperature and the mixture was heated to 37 °C to allow for gelation. Then, 2.0 mL of pre-heated PBS media (37 °C) was added onto the top of the hydrogel. The sample was quickly put into an incubator at 37 °C under gentle shaking. At different time intervals, 150 μL of the supernatants were taken and added into wells of a 96-wells plate. Fluorescence intensities were recorded using a microplate reader (Spectra Max Gemini EM XPS, Molecular Devices, San Jose, CA) at an excitation of 640 nm and an emission of 700 nm.

### 2.9. *In vitro* cell cytotoxicity

Cytotoxicity of the thermally-gelling hydrogels to cells was conducted according to the publication.^39^ Briefly, 0.54 mL of DMEM was added into the thermally-gelling hydrogels and incubated at 37 °C in a cell culture incubator. After culture for 3 days, 0.06 mL was taken out as the conditioned media for the cell viability assay. NIH 3T3 fibroblasts were plated at a density of 25 000 cells per well in a 48-well plate and cultured in DMEM media containing 20% of the above-collected conditioned media. Cell viability was then characterized using a modified Alamar Blue dye assay.^40^

### 2.10. *In vivo* biocompatibility and cancer cell trap

Animal subcutaneous implantation model was used to determine the tissue compatibility of thermally-gelling hydrogel as described previously.^41^ In brief, a 100 μL of the pre-gel nanoparticle dispersion (60 mg ml^−1^) and saline as control was subcutaneously injected in dorsally in a Balb/C mouse (25 g body weight) obtained from The Jackson Laboratory (Bar Harbor, ME). After implantation for 24 hours, the mouse was sacrificed and the implants and the surrounding tissues were isolated, and then sectioned for H&E staining. Additionally, a subcutaneously implanted cancer trap mouse model was used to assess the ability of chemokine-releasing thermal gelling hydrogel for recruiting prostate cancer cells.^42^ 2.0 μL of Epo (10 units in PBS) was mixed with 50 μL of the thermally-gelling nanoparticle dispersion (80 mg mL^−1^ in PBS), and then was injected into both sides of the back of mice *via* 18-gauge needle. After 12 hours, intravascular injection of prostate cancer cells (DAB2IP-knockdown PC3 cells, gift from Dr Jer-Tsong Hsieh at the University of Texas Southwestern Medical Center at Dallas) was administered into mice. The cells were labelled with Vybrant™ DiD Cell-Labeling Solution (Thermo Fisher Scientific, Waltham, MA) according to the manufacturer's protocol before injection. *In vivo* cell recruitment to the sites of hydrogel implants was monitored using Kodak In Vivo Imaging System FX Pro (Carestream Health Inc., New Haven, CT, USA) as described previously.^42^ At the end of the study, implants and surrounding tissues were frozen sectioned and analyzed using H&E and immunohistochemistry as previously described.^43^ Images were taken utilizing a Leica fluorescence microscope (Leica Microsystem GmbH, Wetzlar, Germany) combined with a Retiga-EXi CCD camera (QImaging, Surrey, BC, Canada). Cell number was calibrated using ImageJ software. This study was performed in strict accordance with the AAALAC guidelines – Guide for the care and use of laboratory animals (NRC 2011) and was approved by Animal Care and Use Committee of the University of Texas at Arlington.

### 2.11. Statistical analysis

All the data were evaluated using two-tailed Student *t*-test and presented as mean ± standard deviation. Statistical analyses of all data were performed by a Student *t*-test. The results showed significance when *p* value < 0.05. All tests were conducted in triplicate for statistical analysis.

## 3. Results & discussion

### 3.1. Synthesis and characterization of hydrogel nanoparticles

**Fig. 1:**
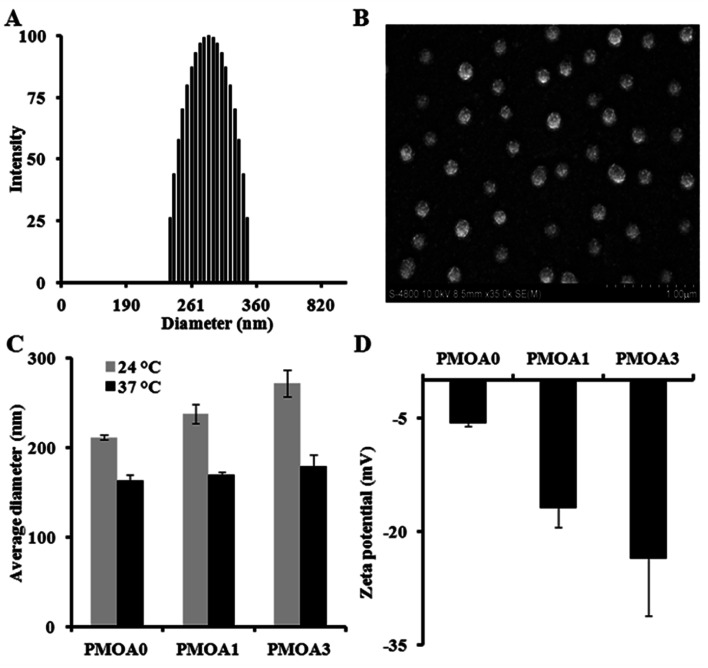
(A) Dynamic Light Scattering (DLS) measurement demonstrating polydispersity of PMOA3 nanoparticle at 24 °C. (B) Scanning Electron Microscope (SEM) image illustrating that the particles are in spherical shape. (C) DLS measurement at both 24 and 37 °C demonstrating an increase in particle size with the increase of acrylic acid content. (D) DLS measurement demonstrating a decrease in zeta potential with the increase of acrylic acid.

Three of hydrogel nanoparticles with various AAc contents were synthesized *via* free radical polymerization (Table 1). All three as-prepared particles are highly mono-dispersed. The polydispersity index of the PMOA3 nanoparticles is 0.005 with narrow size distribution of the nanoparticles at room temperature (Fig. 1A). The finding is in line with the observation of SEM (Fig. 1B). Slight reduction of the particle size in SEM images is due to the particle dehydration during SEM sample preparation. From Fig. 1C, one can observe the dependence of size on temperature for all three nanoparticles. The average diameter for the PMOA0 is 211.4 nm at 24 °C but reduces to 163.3 nm at 37 °C. Similar observation has been obtained on the other two samples. The average diameter is 237.9 nm and 271.8 nm at 24 °C while it is 169.2 nm, and 179.2 nm at 37 °C for PMOA1 and PMOA3, respectively. Furthermore, the average size of the hydrogel nanoparticles increases from 211.4 to 237.9 and 271.8 nm at room temperature with increasing of AAc from 0 to 1 and 3%, respectively. This is because more AAc introduces more charged density into the hydrogel nanoparticles, resulting in greater swelling of the particles. Conductivity titration study reveals that the content of carboxyl groups for PMOA1 and PMOA3 are 9.628 and 23.1 nmole mg^−1^, respectively. The observation can be further confirmed by zeta potential of the hydrogel nanoparticles (Fig. 1D). The zeta potential of the PMOA0, PMOA1 and PMOA3 is −5.69 mV, −16.8 mV to −23.57 mV, respectively. These observations are in good agreement with early publications.^44,45^

### 3.2. Turbidity test and thermally-triggered gelation

**Fig. 2:**
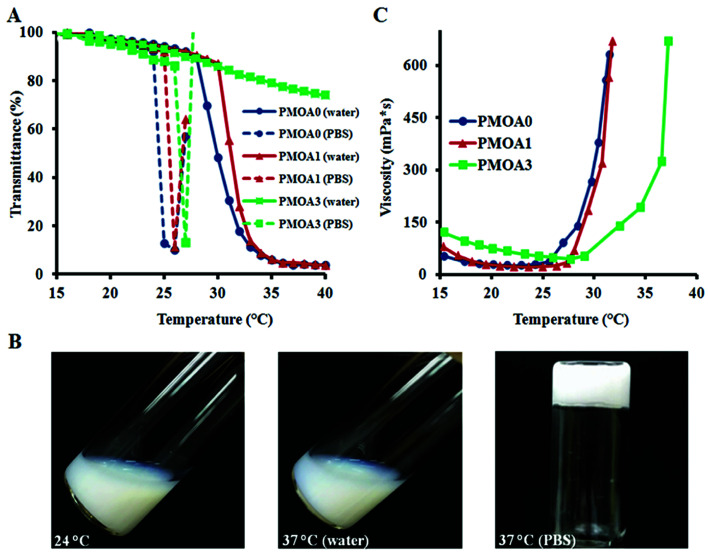
(A) Turbidity measurement of thermogelling nanoparticles (1.0 mg mL^−1^ in deionized water) demonstrating an increase in transition temperature with the increase of acrylic acid. Hydrogel nanoparticles dispersed in PBS (ionic strength: 43.5 mM) have lower transition temperatures than with deionized water. (B) Inversed particle sample (PMOA3) in a test tube illustrating that the hydrogel nanoparticles (60 mg mL^−1^) form gel at physiological temperature. (C) Rheometry measurement of thermogelling nanoparticles (60 mg mL^−1^) demonstrating the viscosity as a function of temperature.

To determine phase transition temperature of the hydrogel nanoparticles, turbidity of the diluted hydrogel nanoparticles (1.0 mg mL^−1^ in DI water and PBS) at different temperatures was determined using a UV-visible spectrophotometer. The results show that all three nanoparticles exhibit temperature-sensitive property (Fig. 2A). For PMOA0 and PMOA1, transmittance of the dispersions decreases gradually with increasing temperature. There is a sharp drop in the transmittance when heated to 28 °C and 30 °C (defined as phase transition temperature), respectively.^46^ On the other hand, onset of the phase transition temperature for PMOA3 cannot be reached even when the temperature is raised up to 40 °C. The increase in the phase transition temperature with increasing AAc contents can be explained as follows. More AAc contributes to high ionic strength (as confirmed in Fig. 1D). In addition, the repulsive force between these negatively charged carboxyl groups keeps the polymer chains from aggregation.^44^ Similar observations are also documented in several recent publications.^44,45^

The phase transition temperatures of these nanoparticles at physiological media (PBS, pH: 7.4 and ionic strength: 43.5 mM) were also investigated (Fig. 2A). One can observe that the phase transition temperature of all three hydrogel nanoparticles shifts to a lower temperature. Furthermore, the presence of salts initiates the nanoparticle's flocculation when temperature is heated above the phase transition temperature. Occurrence of the phenomenon is because the ions in PBS can disturb the closest hydration shells between water and particles, leading to aggregation of the hydrogel particles when heating. On the contrary, all three nanoparticles are highly colloidally stable in DI water at the studied temperature range. Interestingly, all nanoparticles with concentration of 60 mg mL^−1^ exhibit a thermogelling property in PBS media. At room temperature (24 °C), the nanoparticle dispersions can flow freely. However, the dispersions become physically gelled and cannot escape from the inverted tube when temperature reaches to 37 °C (Fig. 2B). This thermally-triggered gelation may be explained as follow: poly[oligo(ethylene glycol) methacrylate] segments of the nanoparticles turns from hydrophilic into hydrophobic beyond the phase transition temperature, leading to the hydrophobic interaction among the particles to form a physical network in presence of salt as shown in recent studies.^24,25^ The thermosensitive hydrogels, PMOA0, PMOA 1 and PMOA3, can revert to sol state by cooling down. This temperature-dependent phase change can be carried out indefinitely at least *in vitro*. Furthermore, the viscosities of the dispersions were measured with increasing temperature. In concurrence with an earlier study, we find a sharp increase in viscosity when the dispersion is heated above the gelation temperature, and the gelation temperature increases with increasing AAc contents (Fig. 2C).^25^

### 3.3. Water loss and microstructure of the hydrogel

**Fig. 3:**
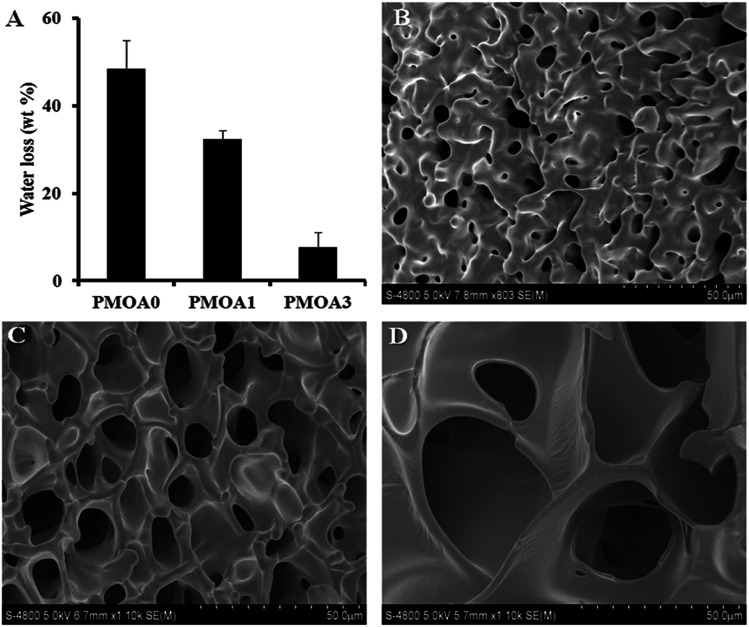
(A) Measurement of water loss demonstrating a decrease in water loss with the increase of acrylic acid. 1.0 mL of hydrogel nanoparticles (60 mg mL^−1^) were incubated at 37 °C for 4 hours, and then the released water was collected from the top of the gels and measured the weight of the lost water. SEM images illustrating porosity of PMOA0 (B), PMOA1 (C), and PMOA3 (D) nanoparticles matrix.

Since the syneresis of hydrogels has adverse effects for applications of the injectable hydrogel scaffold in tissue engineering and drug delivery,^47^ the water loss of the thermogelling macroscopic hydrogels were carried out, and the results are presented in Fig. 3A. Without AAc (PMOA0), approximately 48.4 wt% of water is expelled from the hydrogel after incubated at 37 °C for 4 hours. As expected, addition of AAc has significant impact on water loss. Water loss for PMOA1 (1% AAc) and PMOA3 (3% AAc) were measured to be 32.4 and 7.6 wt%, respectively. The phenomena is in good agreement with recent observations that associate improved hydrogel charges with increased AAc content which in turn, attributes to enlarged pore size, reduced hydrogel shrinkage and augmented water retention of the hydrogel.^21,48^ SEM was further used to investigate microstructure of these hydrogels (Fig. 3B–D). An interconnected porous structure can be observed in all three hydrogels. This structure is very critical for hydrogel scaffolds because it allows cells to migrate, and nutrients and waste products to exchange between the scaffolds and surrounding media.^49^ The presence of AAc makes the pore size of PMOA1 and PMOA3 larger than that of PMOA0 (5.5, 12.3, and 32.1 μm for PMOA0, PMOA1 and PMOA3, respectively). These results are consistent with those of water loss investigation.

### 3.4. *In vitro* release of protein

**Fig. 4:**
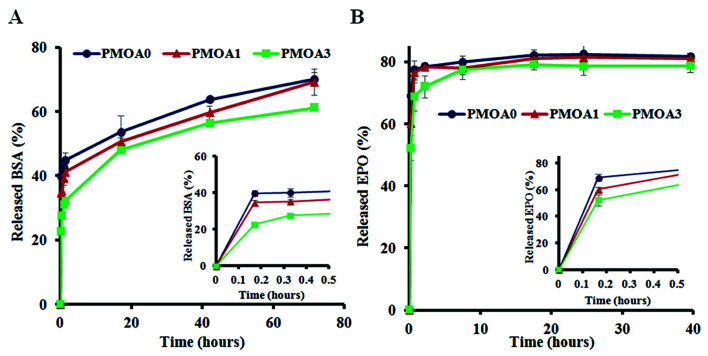
*In vitro* release tests of proteins. (A) Release profiles of Cy®5-labeled BSA and (B) release profiles of Cy®5-labeled Epo.

The slow release properties of hydrogels were first characterized using Cy®5-labeled BSA. We find that all three hydrogels show a burst release at first 1 hour but BSA released from PMOA0 (∼43%) is faster than that of PMOA1(∼39%) and PMOA3 (∼31%). The burst release may create the chemokine gradient for initiating cell migration, while subsequent slow release of BSA maintains the gradient for maximal and accumulative recruitment of the cells. After that, BSA gradually released out from the matrix of these hydrogels. PMOA0 released 50% BSA in 11 hours. On the other hand, the release of 50% BSA from PMOA1 and PMOA3 took longer duration for 17 and 23 hours, respectively (Fig. 4A). With regard to the release of Cy®5-labeled Epo, a similar release profile can be observed (Fig. 4B) although Cy®5-labeled Epo shows a faster release than BSA. The faster release of Epo than BSA may be caused by their size difference. Molecular weights of Epo and BSA are 30 and 66.5 kDa, respectively. It is well established that smaller size of protein has faster release rate.^50^ The surface charge of protein has also been shown to influence protein release from hydrogel nanoparticles.^51^ However, surface charge of proteins play insignificant role in our findings, since both proteins have a similar isoelectric point (around 4.5).

### 3.5. Hydrogel's responses to cell *in vitro* and tissue *in vivo*

**Fig. 5:**
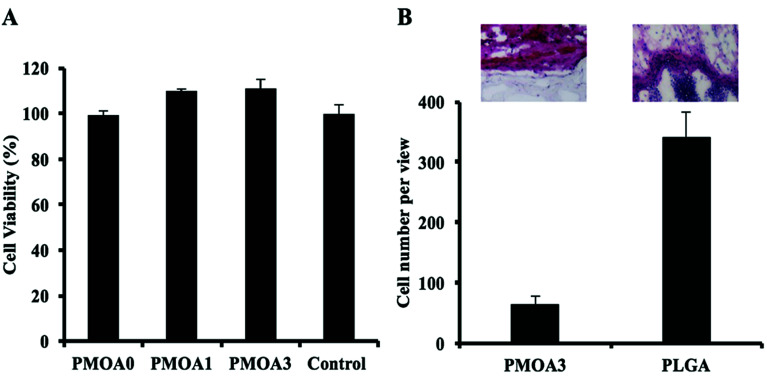
*In vitro* analysis of hydrogel-associated cell and tissue toxicity. (A) *In vitro* toxicity of the thermogelling gels to cells. (B) H&E staining and cell quantification of tissue surrounding PMOA3 and PLGA implants.

To evaluate toxicity of the hydrogels to cell *in vitro*, the conditioned media of the hydrogels were collected at the defined time points, and the toxicity of them to 3T3 fibroblast cells was estimated using an Alamar Blue assay (Fig. 5A). After 3 days, PMOA3 exhibits the least toxic response among these hydrogels although all three hydrogels exhibit minimum toxicity to 3T3 fibroblasts. Combining the results with the protein release *in vitro*, PMOA3 was chosen as a protein delivery scaffold *in vivo*. However, prior to doing that, response of the PMOA3 hydrogel to tissue needs to be further evaluated *in vivo*. Here a mouse subcutaneous implant model was employed, and the poly(lactic-*co*-glycolic acid) (PLGA) particle served as a control.^41^ As expected, PLGA implant triggers more foreign body reactions in the surrounding area of the implant than the PMOA3 gel (Fig. 5B). Quantitative analysis further shows that PMOA3 hydrogel triggers significant lesser inflammatory cell accumulation (∼4×) than the PLGA implants (Fig. 5B). The histological evidence supports that PMOA3 hydrogels possess good tissue compatibility. These results are in consistent with previous finding that poly(oligo(ethylene glycol)) nanoparticles and their derived products have similar tissue responses as the FDA-approved poly(ethylene glycol) polymer.^18^

### 3.6. *In vivo* cancer cell trap

**Fig. 6:**
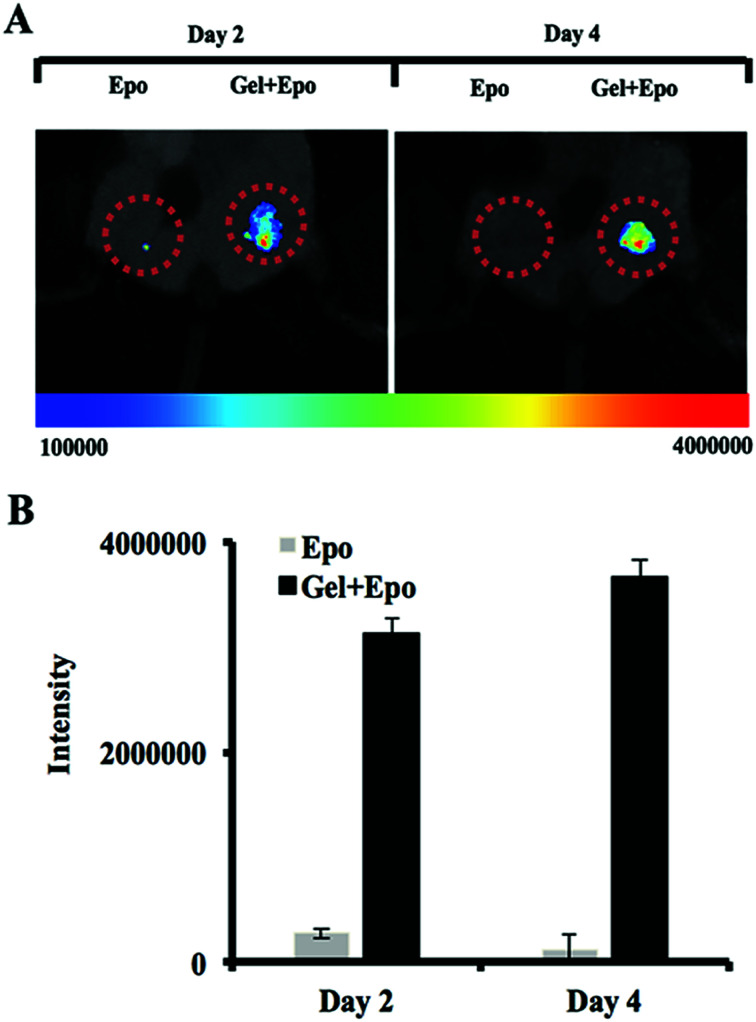
The ability of chemokine-releasing thermogelling nanoparticles to recruit prostate cancer cells in mice. (A) Whole body imaging and (B) fluorescent intensity measurement of NIR-labeled prostate cancer cells at the implant site of Epo and Epo-releasing thermogelling nanoparticles in animals.

To investigate the capability of thermally induced gelling system as a cancer trap implant, Epo-loaded PMOA3 nanoparticles or Epo alone were implanted in the subcutaneous cavity *via* 18-gauge needle. After 12 hours, mice were administered IV injection of DiD-labeled prostate cancer cells (PC3-KD cells). After cancer cell implantation, migratory of the cancer cells were imaged after 2 days and 4 days, respectively. Based on NIR imaging, the Epo-loaded PMOA3 implant recruited significantly more PC3-KD cells than Epo alone (Fig. 6A). Total intensity of PC3-KD cells from the implanted site was 3.14 × 10^6^ at Day 2 and 3.66 × 10^6^ at Day 4 (Fig. 6B). Previous studies have demonstrated that Epo receptor is highly expressed in many different cancer cells such as breast, head-and-neck tumors, colon, lung, prostate and melanoma, and Epo-releasing scaffolds prompts more melanoma cell migration.^42,52–54^ In addition, our previous study has demonstrated that similar hydrogel has controlled protein release properties.^18^ Equally important, using an *in vivo* cancer cell trapping mouse model, Epo-releasing PMOA hydrogel is able to recruit cancer cells to the implant site effectively. This injectable, thermogelling PMOA hydrogel may be used to create Epo gradient for recruiting and trapping circulating cancer cells to reduce or delay cancer metastasis.

## 4. Conclusions

In short, an injectable, thermogelling poly(oligo(ethylene glycol))-based nanoparticles are successfully prepared. Both *in vitro* and *in vivo* testing confirm that the PMOA hydrogels made of these nanoparticles induces minimal toxicity to cells and tissue responses. By simply mixing the protein with the hydrogel nanoparticle at room temperature, the mixture can easily be injected into the body and turn into the physical hydrogel at the body temperature, serving as a depot for controlled release of proteins. *In vivo* cancer cell migration experiment shows a dramatically reduction of metastasis by tuning the release of Epo. In general, this study suggests that this PMOA hydrogel nanoparticle has a great potential in protein therapy and drug delivery.

## Conflicts of interest

Drs Zhou and Tang have a potential research conflict of interest due to a financial interest with Progenitec Inc. A management plan has been created to preserve objectivity in research in accordance with UTA policy.

## Supplementary Material
