# Correction: Preparation of a novel injectable *in situ*-gelling nanoparticle with applications in controlled protein release and cancer cell entrapment

**DOI:** 10.1039/c8ra90099j

**Published:** 2018-12-11

**Authors:** Min Kyung Khang, Jun Zhou, Yihui Huang, Amirhossein Hakamivala, Shuxin Li, Liping Tang

**Affiliations:** Chemistry and Biochemistry Department, University of Texas at Arlington Arlington Texas USA; Bioengineering Department, University of Texas at Arlington P. O. Box 19138 Arlington Texas 76019-0138 USA ltang@uta.edu; Department of Biomedical Science and Environmental Biology, Kaohsiung Medical University Kaohsiung 807 Taiwan

## Abstract

Correction for ‘Preparation of a novel injectable *in situ*-gelling nanoparticle with applications in controlled protein release and cancer cell entrapment’ by Min Kyung Khang *et al.*, *RSC Adv.*, 2018, **8**, 34625–34633.

The authors regret the omission of one of the authors, Shuxin Li, from the original manuscript. The corrected author list is as shown above.

The Royal Society of Chemistry apologises for these errors and any consequent inconvenience to authors and readers.

## Supplementary Material

